# Fine-mapping of the Fusarium head blight resistance QTL *Qfhs.ifa*-*5A* identifies two resistance QTL associated with anther extrusion

**DOI:** 10.1007/s00122-019-03336-x

**Published:** 2019-04-04

**Authors:** Barbara Steiner, Maria Buerstmayr, Christian Wagner, Andrea Danler, Babur Eshonkulov, Magdalena Ehn, Hermann Buerstmayr

**Affiliations:** 1 Department of Agrobiotechnology (IFA-Tulln), Institute of Biotechnology in Plant Production, University of Natural Resources and Life Sciences, Vienna (BOKU), Konrad-Lorenz-Str. 20, 3430 Tulln, Austria; 2 Samarkand Institute of Veterinary Medicine, Samarkand, Uzbekistan

## Abstract

**Key message:**

Fine-mapping separated *Qfhs.ifa-5A* into a major QTL mapping across the centromere and a minor effect QTL positioned at the distal half of 5AS. Both increase Fusarium resistance and anther extrusion.

**Abstract:**

The Fusarium head blight (FHB) resistance QTL *Qfhs.ifa*-*5A* resides in the low-recombinogenic pericentromeric region of chromosome 5A making fine-mapping particularly arduous. *Qfhs.ifa*-*5A* primarily contributes resistance to fungal entry with the favorable allele descending from the highly Fusarium resistant cultivar Sumai-3. Fine-mapping a near-isogenic recombinant inbred line population partitioned the *Qfhs.ifa*-*5A* interval into 12 bins. Near-isogenic lines recombining at the interval were phenotyped for FHB severity, anther retention and plant height. Composite interval mapping separated the initially single QTL into two QTL. The major effect QTL *Qfhs.ifa*-*5Ac* mapped across the centromere and the smaller effect QTL *Qfhs.ifa*-*5AS* mapped to the distal half of 5AS. Although *Qfhs.ifa*-*5Ac* and *Qfhs.ifa*-*5AS* intervals were as small as 0.1 and 0.2 cM, their corresponding physical distances were large, comprising 44.1 Mbp and 49.2 Mbp, respectively. Sumai-3 alleles at either QTL improved FHB resistance and increased anther extrusion suggesting a pleiotropic effect of anthers on resistance. This hypothesis was supported by greenhouse experiments using the susceptible cultivar Remus and its resistant near-isogenic line NIL3 carrying the entire *Qfhs.ifa*-*5A* segment. By manually removing anthers prior to spray inoculation both, Remus and NIL3 became almost equally resistant in the early phase of the disease development and were significantly less diseased than variants without anther manipulation. At late time points the positive effect of the anther removal became smaller for Remus and disappeared completely for NIL3. Results affirm that absence of anthers enhanced resistance to initial infection but did not protect plants from fungal spreading within spikes.

**Electronic supplementary material:**

The online version of this article (10.1007/s00122-019-03336-x) contains supplementary material, which is available to authorized users.

## Introduction

Fusarium head blight (FHB) affects wheat and other small grains throughout the world (McMullen et al. 1997). Epidemics result in significant losses of grain yield and quality but the major concern is the contamination of infected grains with mycotoxins that represent a serious health risk to humans and animal livestock (Dexter and Nowicki 2003; Pestka 2010). Crop management and agrochemical measures are only partly effective making the cultivation of resistant varieties particularly important for disease control (Buerstmayr et al. 2009; Dweba et al. 2017; Parry et al. 1995). FHB resistance is a complex quantitative trait and mainly controlled by small to medium effect QTL; up to now no source of immunity to FHB has been found (Buerstmayr et al. 2009; Schroeder and Christensen 1963). So far, results of more than 120 mapping populations and several association panels have led to the identification of more than 250 quantitative trait loci (QTL) distributed on all 21 wheat chromosomes (Buerstmayr et al. 2009; Jia et al. 2018; Steiner et al. 2017). However, only a few QTL have been validated across studies and solely *Fhb1* (Cuthbert et al. 2006), *Fhb2* (Cuthbert et al. 2007), *Fhb4* (Xue et al. 2010), *Fhb5* (Xue et al. 2011), and *Qfhs.ndsu*-*3AS* (Zhu et al. 2016) have been subjected to fine-mapping to identify tightly linked markers. *Qfhs.ifa*-*5A* (Buerstmayr et al. 2002, 2003) is among the most frequently studied resistance QTL and was validated either individually or in combination with other QTL (Bokore et al. 2017; Chen et al. 2006; Chrpova et al. 2011; Kang et al. 2011; McCartney et al. 2007; Salameh et al. 2011; Suzuki et al. 2012; Tamburic-Ilincic 2012; von der Ohe et al. 2010). *Qfhs.ifa*-*5A* mapped across the centromere of chromosome 5A within a 1.6 cM QTL support interval flanked by the SSR markers *barc186* and *wmc805* (Buerstmayr et al. 2003, 2018). However, based on the IWGSC RefSeq v1.0 (International Wheat Genome Sequencing Consortium [IWGSC] et al. 2018) this interval comprises more than 300 Mbp. Notably, FHB resistance QTL identified in several other germplasms, such as Wangshuibai (Lin et al. 2006), Nyu Bai (Somers et al. 2003), F201R (Shen et al. 2003), Frontana (Steiner et al. 2004), CJ 9306 (Jiang et al. 2007), Haiyanzhong (Li et al. 2011), and Huangfangzhu (Li et al. 2012) overlapped with the *Qfhs.ifa*-*5A* interval. Centromeres and pericentromeric regions are low-recombinogenic intervals and limited or no recombination may be observed that can encompass up to 70% of proximal regions around centromeres (Akhunov et al. 2003; Erayman et al. 2004). As a consequence, fine-mapping and positional cloning of genes near the centromere is extremely challenging. Even so, Xue et al. (2011) was able to narrow down the resistance QTL of Wangshuibai to a 0.3 cM interval and placed *Fhb5* on the short arm between SSR marker *gwm415* and markers *gwm304*, *gwm293*, *barc358*. However, the new refined position still corresponds to more than 110 Mbp, thus reflecting the large discrepancy between genetic and physical distances in centromeric regions.

Different types of resistance have been described (Mesterhazy 1995; Rudd et al. 2001; Schroeder and Christensen 1963). The main resistance types that are frequently evaluated in QTL mapping studies are: (1) resistance to initial infection (type 1) assessed as the incidence of infection after spray or grain spawn inoculation of plants, (2) resistance to fungal spread within the spike (type 2) assessed as the spread of infection within the spike after inoculating single florets and (3) FHB severity or ‘field resistance’, assessed as the overall percentage of symptomatic spikelets after spray or grain spawn inoculation of plants; FHB severity provides an overall estimate of FHB resistance and does not distinguish between specific resistance types. Type 1 and type 2 resistance are under different genetic control (Steiner et al. 2004) and studies showed that *Qfhs.ifa*-*5A* predominantly conditions type 1 and overall FHB severity and to a lesser extent also contributes to type 2 resistance (Buerstmayr et al. 2002, 2003). Plant morphology, especially plant height (Buerstmayr and Buerstmayr 2016; Hilton et al. 1999) and the extent of retained anthers (Buerstmayr and Buerstmayr 2015, 2016; Lu et al. 2013; Skinnes et al. 2010) were found to be particularly associated with type 1 resistance and FHB severity. These morphological traits affect the likelihood for the fungal spores to enter the spikelets and modulate conditions for fungal growth rather than inducing active physiological responses in the host and are considered as passive resistance factors (Mesterhazy 1995). An increase of FHB severity in the presence of retained anthers was reported early (Pugh et al. 1933; Tu 1953). Although the importance of anthers is widely recognized their impact on resistance was seldom included in FHB resistance analyzes. Recently several studies focused on the relationship between FHB resistance and anthers and all observed an increase of infection in the presence of retained anthers (Graham and Browne 2009; He et al. 2014; Kubo et al. 2013a, b; Miller et al. 2004). QTL for anther extrusion and its complement anther retention frequently coincided with QTL for FHB resistance traits (Buerstmayr and Buerstmayr 2015, 2016; Lu et al. 2013; Skinnes et al. 2010). This lead to the conclusion that some of the FHB resistance QTL reported previously in numerous papers could possibly be caused by differences in anther extrusion (Lu et al. 2013).

High-resolution mapping of the pericentromeric region of chromosome 5A identified 70 lines that recombined in the *Qfhs.ifa*-*5A* interval (Buerstmayr et al. 2018). These NILs were phenotyped for FHB resistance in field trials to precisely locate the QTL and pave the way for the ultimate goal of positional cloning resistance underlying gene(s). Additionally we investigated associated morphological traits and their contributions on disease development to gain a better understanding of the mechanism and complexity of the *Qfhs.ifa*-*5A* resistance. In this regard we particularly examined the effect of anthers on FHB resistance of the highly susceptible Remus and its near-isogenic line NIL3 (resistant, carrier of *Qfhs.ifa*-*5A*) by manually removing or manually retaining anthers post-anthesis with following *F. graminearum* spray inoculations.

## Materials and methods

### Field trials

#### Plant material

A doubled haploid (DH) population of the cross Remus/CM-82036 was developed as described by Buerstmayr et al. (2002) and one FHB resistant DH line, selected for the presence of *Fhb1* and *Qfhs.ifa*-*5A* resistance alleles, was five times backcrossed to the cultivar Remus to generate NILs as described in Schweiger et al. (2013). Remus (Sappo/Mex//Famos), a spring wheat cultivar developed by the Bavarian State Institute for Agronomy in Freising, Germany, is highly susceptible to FHB. CM-82036 (Sumai-3/Thornbird-S, donor of *Fhb1* and *Qfhs.ifa*-*5A* resistance allele) is highly resistant to FHB. NIL1 (*Fhb1*, *Qfhs.ifa*-*5A*) and NIL2 (*Fhb1*) were crossed to develop a near-isogenic recombinant inbred line (NI-RIL) population. NIL1 and NIL2 have the same genetic background and differ only for the resistant/susceptible allele at the *Qfhs.ifa*-*5A* locus. Of this cross, 3650 F_2_ plants were genotyped resulting in the identification of 70 NI-RILs comprising 22 distinct haplotypes (Buerstmayr et al. 2018). Identified recombinant plants were advanced to the F_3_ generation to select for homozygous NI-RILs and propagated for multi-environment testing. All selected NI-RILs, along with Remus, CM-82036 and six additional Remus NILs that either had the *Qfhs*-*ifa*-*5A* interval introgressed (NIL1, NIL3, NIL30) or Remus alleles at the QTL interval (NIL2, NIL4, NIL27) were tested in field trials at IFA-Tulln (latitude 48°20′0″N, longitude 16°3′0″E, altitude 177 m) from 2014 to 2017. Experiments were arranged in a randomized complete block designs with four blocks in year 2014, 2015 and 2016, and two blocks in year 2017. Sowing time was beginning of March in all years. Blocks (two at a time in 2014–2016) were sown 1–2 weeks apart depending on the specific weather conditions, which delayed anthesis by 1–3 days for the later-sown blocks. Plots consisted of double rows of 0.7 m length with 17 cm row spacing. The number of double rows arranged side-by-side in each block were 9–12, depending on the specific experiments; the sizes of the individual blocks in the field ranged from 6 m × 8 m to 4.5 m × 10 m. Seed treatment, sowing density and crop management were essentially the same as described by Buerstmayr et al. (2002).

#### Fusarium inoculation and trait assessments

Lines were evaluated for FHB incidence, FHB severity, anther retention and plant height. To ensure successful FHB infection and disease development, artificial spray inoculation following mist irrigation was performed. Entire plots of individual blocks were inoculated when the earliest plot of a block reached mid-anthesis and was repeated every other day until 2 days after the last plot of the block reached mid-anthesis, resulting in up to five inoculum applications per plot. A macroconidial suspension of the *F. culmorum* single-spore isolate ‘Fc91015’ was prepared as described by Buerstmayr et al. (2000) and aliquots were stored at − 80 °C. Just prior to inoculation the required amount of inoculum aliquots was thawed in lukewarm water and diluted to the desired macroconidia concentration of 2.5 × 10^4^ ml^−1^ using tap water. About 100 ml m^−2^ of the freshly diluted conidia suspension were sprayed onto the heads using a battery-driven backpack sprayer. Inoculations were carried out in the late afternoons from 4:00 to 6:00 p.m. An automated mist-irrigation system triggered by leaf wetness measurement maintained humidity and kept plants wet during the first 20 h after inoculation.

FHB incidence was reported as the percentage of heads showing ≥ 1 symptomatic spikelets out of 40 randomly chosen heads per plot in year 2014, 2015 and 2016. Within each experiment, all plots were individually scored at a specific scheduled date post-anthesis when symptoms were clearly visibly. Depending on environmental conditions of the respective year, scoring date ranged from 21 to 24 days post-anthesis. FHB severity was visually estimated as the percentage of infected spikelets within each plot on days 10, 14, 18, 22 and 26 after anthesis. The area under the disease progress curve (AUDPC) was calculated as described by Buerstmayr et al. (2000) and used as an integrated measure of intensity and progress of FHB disease severity from 10 to 26 days after anthesis. Percentage of retained anthers (AR) was assessed on 20 florets of five randomly chosen heads 5 days after anthesis. The basal florets of four spikelets in the central part of each head were manually opened and characterized as retained as soon as one or more anthers remained within the floret or were trapped between lemma and palea. Plant height (PH) was measured in years 2014, 2015 and 2017.

#### Statistical analyses

Statistical analyses of field data were performed in R version 3.4.3 (R Core Team 2018). For analysis of variance, the lme4 package (Bates et al. 2015) was used applying following model:

$$t_{ijk} = \mu + g_{i} + y_{j} + \left( {gy} \right)_{ij} + b_{k} \left( {y_{i} } \right) + e_{ijk} ,$$

where *t*_*ijk*_ is the phenotypic value for the *i*th genotype in the *k*th block of the *j*th year; *µ* is the intercept term, *g* is the effect of the genotype, *y* is the effect of the year, *gy* is the interaction effects between genotype and year, *b*(*y*) the block within year effect and *e* is the residual term. Variance components were determined by the restricted maximum likelihood (REML) method assuming a random effects model. Heritability coefficients were estimated from variance components with the equation

$$H^{2} = \sigma_{G}^{2} /\left( {\sigma_{G}^{2} + \, \sigma_{GxY}^{2} /y \, + \, \sigma_{E}^{2} /yr} \right),$$

where $\sigma_{G}^{2}$ is the genotypic variance, $\sigma_{GxY}^{2}$ the genotype by year interaction, $\sigma_{E}^{2}$ the residual variance, *y* the number of years, and *r* the number of replications (Nyquist and Baker 1991) where all effects were considered random. Pearson correlation coefficients of individual traits were estimated for all pairwise experiment combinations and correlations between FHB severity, FHB incidence, AR and PH were estimated for individual years and for means over all experiments.

Genotypic data and genetic map were the same as described in Buerstmayr et al. (2018). Briefly, screening 3650 F_2_ NILs for recombinations between the QTL flanking markers identified 70 plants having a recombination in the target interval. These 70 NI-RILs were genotyped with 28 markers. Markers were selected according their assignment to chromosome 5A using CS-N5AT5B (Sears 1954), and cytogenetic Chinese Spring deletion lines C-5AS1-0.40, 5AS3-0.75 and 5AS6-0.97 (Endo and Gill 1996). Markers partitioned the 5A linkage group into 15 subintervals for a total length of 18.3 cM (10.6 cM on 5AS, 7.7 cM on 5AL). The QTL interval of *Qfhs.ifa*-*5A* spanned across the centromere and covered a distance of 2.5 cM.

QTL analyses were carried out on Qgene (version 4.2.3) mapping software (Joehanes and Nelson 2008) on NIL means for individual experiments and for means across experiments. QTL search was conducted on a 0.1 cM scan intervals using composite interval mapping (CIM) of the least-squares method of Zeng (1994). QTL were selected as cofactors for background control set by a forward regression approach. A permutations test applying 1000 iterations determined the critical LOD values at a type I error rate of *α* < 0.05 and *α* < 0.01 for each trait and experiment. The percentage of phenotypic variance (PV%) explained by a QTL was determined by the partial correlation coefficient and additive effects were calculated. NILs were grouped by their QTL improving alleles and Tukey’s multiple range test at *p* < 0.05 was used to conduct pairwise group comparisons of mean trait performances. Linkage groups and LOD profiles were drawn with MapChart v2.2 (Voorrips 2002). Marker sequences of all mapped markers were blast searched against the IWGSC RefSeq v1.0 to obtain their physical positions (Alaux et al. 2018; International Wheat Genome Sequencing Consortium [IWGSC] et al. 2018). Gene annotation of the refined QTL interval was then retrieved from IWGSC RefSeq v1.0 (https://urgi.versailles.inra.fr/download/iwgsc/IWGSC_RefSeq_Annotations/v1.0/).

### Greenhouse trials

#### Plant material

Two genotypes were used in greenhouse experiments to further analyze the association of FHB resistance conferred by *Qfhs.ifa*-*5A* and anther extrusion, the susceptible spring wheat cultivar Remus and its near-isogenic line NIL3 possessing *Qfhs.ifa*-*5A* in a 98.5% Remus background (Schweiger et al. 2013).

#### Fusarium inoculation and trait assessments

In March and September 2015 seeds of Remus and NIL3 were germinated in trays on a mixture of re-cycled compost and sand and vernalized at 4 °C, 12 h day/night light regime, for 2 weeks. Five seedlings of one genotype were transplanted into pots (20 cm diameter, 20 cm height) filled with 4 l of potting soil (a mixture of heat-sterilized compost, peat, sand and rock flour) and placed in the greenhouse. Three experiments were planted with 225 plants (45 pots, 5 plants per pot) per genotype and experiment, whereas experiment 1 was conducted in spring 2015 and experiments 2 and 3 in fall 2015. The experimental design was a split plot design, with six blocks, and 7 to 8 pots per genotype were grouped together in two rows within blocks. Temperature in the greenhouse was 18/12 °C (day/night) from tillering to heading with 12–14 h of light. During flowering time the conditions in the greenhouse were set at 22/18 °C (day/night) with a 16-h photoperiod at 15,000 lx.

Before anthesis spikes were trimmed to 12 spikelets (experiment 1) and 16 spikelets (experiment 2, 3) by cutting off the least developed basal and apical spikelets. The possible contribution of anthers to FHB infection was compared by manipulating spikes post-anthesis (GS69 anthesis completed) and before *F. graminearum* spray inoculation. (1) Anthers of all spikelets were detached from the filaments and discarded (anthers removed); (2) anthers of the two basal florets for all spikelets per head were detached and immediately replaced back between palea and lemma. Thereby anthers remained inside the floret and could not be extruded (anthers retained); (3) Control heads without anther manipulation. One day after anther manipulation primed and control spikes were spray inoculated with a *F. graminearum* conidia suspension produced as described in Schweiger et al. (2016). Two ml inoculum per spike (conidia concentration of 2 × 10^4^ ml^−1^ and 1 ml Tween 20 per liter) was applied using a hand held sprayer. Inoculated spikes were covered with plastic bags for 24 h to assure high humidity for fungal development. Anther manipulation and Fusarium spray inoculations were performed on alternate days.

Disease incidence and disease severity were evaluated by determining either the number of infection sites or the total number of symptomatic spikelets per spike. Number of infection sites was assessed at 6, 10 and 14 days after inoculation (dai) and the percentage of infection sites per spike was calculated as a measure for disease incidence, whereas the total number of infected spikelets per spike was monitored over five time points (6, 10, 14, 18 and 22 dai) and the percentage of infected spikelets per spike represents disease severity.

#### Statistical analysis

Variance components were determined applying following linear mixed effects model:

$$Y_{ijklmn} = \mu + g_{i} + t_{j} + gt_{ij} + e_{k} + ge_{ik} + te_{jk} + gte_{ijk} + b_{l} (e_{k} ) + r_{m} \left[ {be} \right]_{lk} + p_{n} \left( {rbe} \right)_{mlk} + \varepsilon_{jklmn}$$

where µ denotes the overall mean, *g*_*i*_ the genetic effect of genotype *i*, *t*_*j*_ the effect of treatment *j*, *gt*_*ij*_ the interaction between genotype *i* and treatment *j*, *e*_*k*_ the effect of the experiment *k*, *ge*_*ik*_ the interaction between genotype *i* and experiment *k*, *te*_*jk*_ the interaction between treatment *j* and experiment *k*, *gte*_*ijk*_ the interaction between genotype *i*, treatment *j*, and experiment *k*, *b*_*l*_(*e*_*k*_) the effect of the block *l* nested in the experiment *k*, *r*_*m*_[*be*]_*kl*_ the effect of the row *m* nested in the block *l* of experiment *k*, *p*_*n*_[*rbe*]_*mlk*_ the effect of the pot *n* nested in the row *m* of the block *l* in the experiment *k* and *ε*_*jklmn*_ the residual effect. Genotype, treatment and genotype-by-treatment interaction were considered as fixed effects, all other factors were considered as random effects.

Plants were grouped according to their genotype-by-treatment combination comprising Remus and NIL3 variants having either all anthers manually removed, manually retained or remained unmanipulated. Fusarium resistance traits of all groups were compared using Tukey’s multiple range test at *p* < 0.01.

## Results

### Field trials

#### Phenotypic results

Among all NILs tested, the resistant parent NIL1 (*Fhb1*, *Qfhs.ifa*-*5A*) performed best for FHB resistance traits and had the lowest proportion of retained anthers. NIL3 (*Qfhs.ifa*-*5A*) was almost as resistant as NIL1 and had only slightly more anthers retained. NIL2 (*Fhb1*) and Remus were highly diseased and had approximately more than four times higher AUDPC scorings, close to two times higher FHB incidence and more than two times higher proportion of retained anthers (Table 1). NIL1, NIL2, NIL3 and Remus had similar PHs.

**Table 1:** Overall means of Remus and NIL1, NIL2, NIL3, means and ranges of the near-isogenic recombinant inbred line (NI-RIL) population for individual years and across years, variance component estimates of genotype ($\sigma_{\text{Genotype}}^{2}$), genotype-by-year interaction ($\sigma_{{{\text{Genotype}}*{\text{Year}}}}^{2}$), least significant difference (LSD α 0.05%) and heritability coefficients (*H*^2^) for FHB severity, FHB incidence, anther retention and plant height

	FHB severity (AUDPC)	FHB incidence (%)	Anther retention (%)	Plant height (cm)
Remus	475.4	81.4	58.6	86.3
NIL1^a,b^	95.1	48.8	22.9	87.2
NIL2^a,b^	455.2	85.2	63.1	86.1
NIL3^a^	136.4	56.6	26.1	88.8
*NI-RIL population*				
Overall mean (range)	294.3 (113.5–595.6)	68.9 (48.8–91.3)	44.4 (21.0–66.7)	86.3 (80.8–91.0)
2014 (range)	171.3 (50.3–517.2)	43.8 (20.6–74.4)	42.1 (17.5–67.5)	87.8 (82.5–92.5)
2015 (range)	341.5 (106.3–743.4)	75.8 (49.4–97.5)	44.8 (13.8–73.8)	–
2016 (range)	377.9 (161.5–721.0)	83.4 (66.3–96.3)	45.9 (22.0–68.8)	85.1 (77.5–90.0)
2017 (range)	66.7 (7.2–213.5)	–	43.6 (12.5–72.5)	86.2 (82.5–90.0)
Variance ± SD				
$\sigma_{\text{Genotype}}^{2}$	14,686 ± 121.19	109.32 ± 10.45	121.10 ± 11.00	2.23 ± 1.49
$\sigma_{\text{Genotype*Year}}^{2}$	3162 ± 56.23	18.53 ± 4.30	7.88 ± 2.80	0.35 ± 0.59
*H*^2^	0.92	0.88	0.91	0.67
LSD 5%	73.78	8.61	10.34	2.75

^a^Near-isogenic lines of Remus [NIL1 (*Fhb1* + *Qfhs.ifa*-*5A*), NIL2 (*Fhb1*), NIL3 (*Qfhs.ifa*-*5A*)]

^b^Parents of the near-isogenic recombinant inbred line (NI-RIL) population

**Fig. 1:**
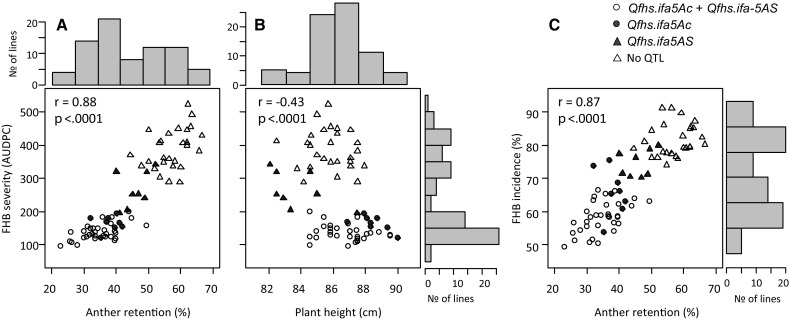
Scatterplots with marginal histograms of overall means for FHB severity (AUDPC) against anther retention (**a**) and plant height (**b**) and for FHB incidence (**c**) against anther retention (%). QTL status at *Qfhs.ifa5*-*Ac* and *Qfhs.ifa*-*5AS* of individual NILs is indicated by different symbols

FHB severity, FHB incidence and AR all displayed a bimodal frequency distribution and PH showed a normal distribution (Fig. 1). Disease levels differed among years and were highest in 2015 and 2016, followed by 2014. A substantially lower disease level was observed in the year 2017 because of cooler night temperatures (also below 15 °C) during inoculation period. This reduced FHB infection and slowed down disease development leading to a very low disease pressure. The extent of AR and PH levels varied only slightly between experiments. For all traits, $\sigma_{\text{Genotype}}^{2}$ was a main source of variance and was much higher than $\sigma_{{{\text{Genotype}}*{\text{Environment}}}}^{2}$ interactions leading to high heritability coefficients of *H*^2^ 0.92, 0.88, 0.91 and 0.67 for FHB severity, FHB incidence, AR and PH, respectively (Table 1). A strong positive correlation was observed between AR and the Fusarium resistance traits FHB severity (*r* = 0.88) and FHB incidence (*r* = 0.87) for means across years and were all significant at *p* < 0.0001 in individual and across years. PH showed a moderate to weak negative correlation to FHB severity (*r* = −0.43, *p* < 0.0001), FHB incidence (*r *= −0.44, *p* < 0.0001) and AR (*r *= −0.42, *p* = 0.0002) for means across years, and no relationship in year 2017. A lower proportion of retained anthers strongly increased FHB resistance (Fig. 1a), and taller plants improved resistance and decreased AR (Fig. 1b, c). Generally, between-experiments correlations were high for FHB severity (*r* = 0.81–0.89), FHB incidence (*r* = 0.75–0.85), and AR (*r* = 0.69–0.78) and moderate for PH (*r* = 0.40–0.46). Pearson correlation coefficients of individual traits for all pairwise experiment combinations and between traits for individual years and across years are provided as Supplementary Table S1 and Fig. S1.

### Fine-mapping of *Qfhs.ifa*-*5A*

**Fig. 2:**
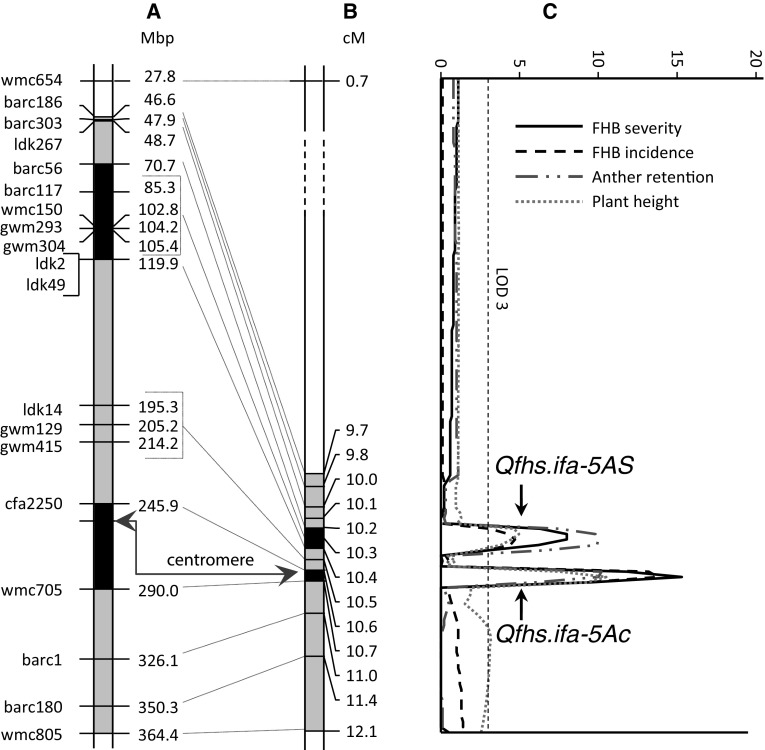
Genetic and physical map of the wheat chromosome 5A. **a** Mbp positions of markers are derived from IWGSC RefSeq v1.0, **b** genetic linkage map of NI-RIL population (Buerstmayr et al. 2018), **c** QTL graphs for FHB severity (AUDPC), FHB incidence (%), anther retention (%) and plant height for means across experiments. Highlighted gray intervals in chromosome bars refer to the initial *Qfhs.ifa*-*5A* support interval. Highlighted black intervals refer to the fine-mapped *Qfhs.ifa*-*5AS* (distal) and *Qfhs.ifa*-*5Ac* (centromeric) support interval. Position of centromere is indicated

The initial QTL interval covered a 1.6 cM genetic distance and was flanked by *barc186* and *wmc805*. Blast search against the IWGSC RefSeq v1.0 placed *barc186* and *wmc805* to positions 46.6 and 364.4 Mbp, respectively. Hence, the 1.6 cM interval corresponded to a 317.8 Mbp physical distance. Genetically, fine-mapping separated this interval into 12 bins, eight on the short, three on the long arm and one across the centromere (Fig. 2). *Cfa2250* (at 245.9 Mbp) and *wmc705* (at 290 Mbp) were the most proximal markers on the 5A short and long arm, respectively.

Composite interval mapping of *Qfhs.ifa*-*5A* against the saturated genetic map separated the initially reported single QTL into a major and a minor effect QTL (Fig. 2a). The major QTL mapped across the centromere being flanked by marker *cfa2250* on the short arm and *wmc705* on the long arm. The second, smaller effect QTL mapped more distal and was flanked by markers *ldk49* and *barc56* and the QTL peak fell to a cluster of four co-segregating markers (*gwm304*, *gwm293*, *wmc150*, and *barc117*). In the following, the proximal QTL is referred to as *Qfhs.ifa*-*5Ac* and the more distal QTL as *Qfhs.ifa*-*5AS*. QTL were delimited to genetic intervals as small as 0.1 and 0.2 cM, respectively, with their QTL peaks being separated by 0.3 cM only. Based on the Mbp position of markers on the IWGSC RefSeq v1.0 and on centiRay (cR) positions on the radiation hybrid map reported by Buerstmayr et al. (2018), *Qfhs.ifa*-*5Ac* and *Qfhs.ifa*-*5AS* mapped to physically distant regions placing all markers of *Qfhs.ifa*-*5AS* to the distal half of 5AS and markers of *Qfhs.ifa*-*5Ac* in proximity of the centromere with their QTL peaks being more than 140 Mbp apart (Fig. 2a). Although the QTL intervals were genetically very small, the corresponding physical intervals were large and comprised 44.1 for *Qfhs.ifa*-*5Ac* and 49.2 Mbp for *Qfhs.ifa*-*5AS*. According to the gene annotation of the IWGSC RefSeq v1.0, assembly of Chinese Spring 101 high confidence (HC) and 248 low confidence (LC) genes are located in the *Qfhs.ifa*-*5Ac* interval, and 240 HC and 351 LC in the interval of *Qfhs.ifa*-*5AS*.

The major QTL *Qfhs.ifa*-*5Ac* was significantly associated with FHB resistance traits across years as well as in all individual years. It explained for FHB severity and FHB incidence between 42 to 60% and 36 to 58% of the phenotypic variance with LOD scores ranging from 8.2 to 15.3 and from 7.5 to 14.5, respectively (Table 2). Similarly, the minor QTL *Qfhs.ifa*-*5AS* was significantly associated with FHB resistance traits, though results of individual experiments varied in their effects and were not significant for FHB incidence in year 2014. Generally, *Qfhs.ifa*-*5AS* was more effective in years with higher disease levels and accounted for > 30% of the phenotypic variance for FHB severity in year 2015 (LOD 7.4) and 2016 (LOD 6.2), and 25% for FHB incidence in 2015 (LOD 4.8) (Table 2). NIL1 (carrier of the Sumai-3 derived resistance) contributed the resistance improving alleles for *Qfhs.ifa*-*5Ac* and *Qfhs.ifa*-*5AS.*

**Table 2:** Position and estimates of *Qfhs.ifa*-*5Ac* and *Qfhs.ifa*-*5AS* for FHB severity, FHB incidence, anther retention and plant height using composite interval mapping (CIM)

QTL	*Qfhs.ifa*-*5Ac*			*Qfhs.ifa*-*5AS*		
Flanking markers	*cfa2250*–*wmc705*			*barc56*–*ldk49*		
Position	10.6–10.7 cM			10.2–10.4 cM		
	LOD^a^	PV%^b^	Add^c^	LOD^a^	PV%^b^	Add^c^
FHB severity (AUDPC)^d^						
Across years	**15.3**	0.60	− 76.4	**8.0**	0.39	− 47.8
2014	**8.2**	0.42	− 69.9	**2.6**	0.16	− 40.5
2015	**11.6**	0.51	− 109.4	**7.4**	0.36	− 80.9
2016	**12.6**	0.53	− 90.8	**6.2**	0.31	− 60.7
2017	**13.2**	0.55	− 40.2	*1.9*	0.11	− 14.9
FHB incidence (%)^d^						
Across years	**13.8**	0.57	− 6.8	**4.7**	0.25	− 3.5
2014	**9.3**	0.47	− 10.1	n.s.	–	–
2015	**14.5**	0.58	− 8.9	**4.8**	0.25	− 4.5
2016	**7.5**	0.36	− 4.7	*2.3*	0.13	− 2.5
Anther retention (%)^d^						
Across years	**10.2**	0.46	− 6.1	**10.0**	0.45	− 5.7
2014	**4.6**	0.27	− 5.6	**3.6**	0.22	− 5.3
2015	**5.8**	0.30	− 6.6	**7.4**	0.36	− 7.8
2016	**5.3**	0.28	− 5.8	**4.4**	0.24	− 6.1
2017	**5.6**	0.29	− 6.5	**4.1**	0.22	− 6.4
Plant height (cm)^e^						
Across years	**10.5**	0.47	1.5	**5.0**	0.26	− 1.0
2014	**5.1**	0.29	1.6	*2.3*	0.14	− 1.0
2016	**9.3**	0.43	1.9	**4.5**	0.24	− 1.4
2017	**3.7**	0.20	1.2	*2.4*	0.13	− 0.9

^a^Significance threshold for LOD values were obtained by permutation tests (1000 iterations) for each experiment and trait; LOD values > *α* 0.01 are in bold; LOD values > *α* 0.05 are in italic; n.s. = non-significant

^b^Percentage of phenotypic variance explained by the QTL

^c^Negative additive effects denote trait-decreasing effect of the Sumai-3 alleles

^d^Cofactor selection for CIM analyses: *gwm304*, *cfa2250* and *barc100*

^e^Cofactor selection for CIM analyses: *gwm304*, *cfa2250*

**Fig. 3:**
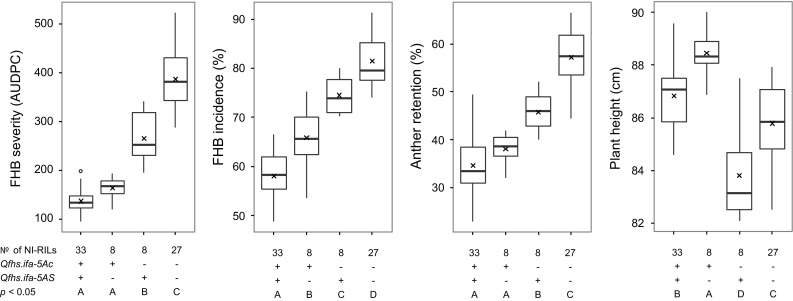
Boxplots of NI-RILs grouped by their resistance status at *Qfhs.ifa*-*5Ac* and *Qfhs.ifa*-*5AS* for means across experiments of FHB severity, FHB incidence, anther retention (%), and plant height. Solid bold lines and crosses indicate the medians and the means, respectively. Means of groups with different letters are significantly different at *p* < 0.05 using Tukey’s multiple range test

Both of the two QTL were significantly associated with AR and PH in all experiments and for the overall means across experiments (Figs. 2c, 3; Table 2). The alleles of NIL1 decreased the proportion of retained anthers for both QTL, with LOD scores ranging from 4.6 to 10.2 and from 3.6 to 10 for *Qfhs.ifa*-*5Ac* and *Qfhs.ifa*-*5AS*, respectively. Although explained phenotypic variance and LOD scores for PH were relatively high for *Qfhs.ifa*-*5Ac* and smaller for *Qfhs.ifa*-*5AS*, the corresponding additive effects were rather small. The NIL1 allele increased PH at *Qfhs.ifa*-*5c* by approximately 1.5 cm, while it reduced PH by about 1 cm at *Qfhs.ifa*-*5AS* (Table 2).

NI-RILs were subdivided into four subgroups contrasting for their resistance combinations for either one or both QTL at *Qfhs.ifa*-*5Ac* and *Qfhs.ifa*-*5AS.* Overall means of individual groups and results of pairwise comparisons using Tukey’s multiple range tests are summarized in Table 3, and boxplots illustrate their distributions (Fig. 3). The ranking of the subgroups was constant in all experiments, with NILs carrying favorable alleles at both QTL being most resistant and having most anthers extruded, closely followed by and in most cases not significantly different from NILs having the positive alleles at *Qfhs.ifa*-*5c* only. The *Qfhs.ifa*-*5c* subgroup was in all experiments significantly more resistant than NILs containing the positive alleles only at *Qfhs.ifa*-*5AS* and had fewer anthers retained in all experiments, although these differences were not always significant. The *Qfhs.ifa*-*5AS* subgroup was more resistant and had fewer anthers retained compared to the subgroup of NILs carrying the unfavorable alleles at either QTL. These differences were significant in most, though not all experiments. The positive allele at *Qfhs.ifa*-*5Ac* increased PH, while it decreased PH at *Qfhs.ifa*-*5AS,* accordingly subgroups contrasting at one of the two QTL significantly differed for PH, whereas differences to subgroups having none or both resistance QTL were less pronounced and not always significant.

**Table 3:** Means and standard deviations (SD) of overall means across experiments for NI-RILs grouped by their resistance status at *Qfhs.ifa*-*5Ac* and *Qfhs.ifa*-*5AS* for FHB severity, FHB incidence, anther retention and plant height

Trait	*Qfhs.ifa*	*Qfhs.ifa*	*N* ^a^	Across years	2014	2015	2016	2017
	*5Ac*	*5AS*		Mean (± SD)	Mean (± SD)	Mean (± SD)	Mean (± SD)	Mean (± SD)
FHB severity (AUDPC)^b^								
	+	+	33	136.6 (± 24.5)^a^	87.0 (± 24.1)^a^	182.1 (± 42.8)^a^	253.4 (± 60.1)^a^	22.7 (± 7.9)^a^
	+	−	8	163.5 (± 23.1)^a^	82.1 (± 28.1)^a^	238.6 (± 34.2)^a^	283.5 (± 67.1)^a^	18.5 (± 9.7)^a^
	−	+	8	265.6 (± 55.0)^b^	189.1 (± 56.7)^b^	357.2 (± 101.1)^b^	417.4 (± 70.8)^b^	90.9 (± 39.8)^b^
	−	−	27	387.0 (± 60.3)^c^	297.0 (± 93.3)^c^	562.3 (± 108.1)^c^	546.3 (± 70.0)^c^	127.5 (± 40.3)^c^
FHB incidence (%)								
	+	+	33	58.1 (± 4.8)^a^	32.3 (± 7.4)^a^	63.8 (± 7.6)^a^	77.2 (± 5.8)^a^	–
	+	−	8	65.8 (± 7.0)^b^	33.3 (± 6.6)^a^	70.2 (± 4.7)^b^	79.1 (± 7.8)^a^	–
	−	+	8	74.5 (± 4.1)^c^	51.9 (± 7.4)^b^	82.0 (± 7.7)^c^	86.5 (± 3.6)^b^	–
	−	−	27	81.4 (± 4.9)^d^	59.0 (± 8.8)^b^	90.2 (± 3.7)^d^	91.5 (± 2.6)^b^	–
Anther retention (%)								
	+	+	33	34.6 (± 5.9)^a^	33.5 (± 8.5)^a^	34.1 (± 8.2)^a^	36.5 (± 7.7)^a^	34.3 (± 8.4)^a^
	+	−	8	38.1 (± 3.3)^a^	35.0 (± 5.4)^ab^	39.4 (± 6.0)^a^	44.4 (± 4.3)^ab^	33.1 (± 4.6)^a^
	−	+	8	45.8 (± 4.3)^b^	43.9 (± 6.6)^b^	41.9 (± 7.7)^a^	51.7 (± 8.9)^bc^	45.6 (± 7.0)^b^
	−	−	27	57.2 (± 5.5)^c^	54.4 (± 6.2)^c^	60.4 (± 8.7)^b^	56.1 (± 7.7)^c^	57.6 (± 8.6)^c^
Plant height (cm)								
	+	+	33	86.8 (± 1.4)^b^	88.5 (± 1.9)^a^	–	85.7 (± 1.6)^ab^	86.3 (± 2.5)^ab^
	+	−	8	88.5 (± 0.9)^a^	90.8 (± 1.4)^a^	–	87.5 (± 1.8)^a^	88.1 (± 1.8)^a^
	−	+	8	83.8 (± 1.8)^d^	85.7 (± 2.1)^b^	–	81.7 (± 2.7)^c^	84.4 (± 1.8)^b^
	−	−	27	85.8 (± 1.3)^c^	87.0 (± 2.0)^b^	–	84.6 (± 1.7)^b^	85.9 (± 2.0)^ab^

QTL classes with different index letters are significantly different at *p* < 0.05 based on Tukey’s multiple range test

^a^*N* number of lines per genotypic group, ^b^*AUDPC* area under the disease progress curve

### Green house trials simulating anther extrusion/retention

FHB infections were observed for all analyzed variants in all experiments comprising Remus and NIL3 with manually removed or retained anthers and unmanipulated control spikes. In each of the three individual experiments, 61 to 75 heads per genotype-by-treatment variant were analyzed resulting in a total of 192 to 210 heads per variant across all experiments (Table S2). FHB incidence (measured as percentage infections sites) could only be determined unambiguously at dai(6) and dai(10), at later time points incidence scoring was confounded by fungal spreading. Scores of FHB incidence and FHB severity (measured as percentage of symptomatic spikelets) at dai(6) and dai(10) were almost identical, thus in the following only FHB severity results are reported.

ANOVA resulted in significant effects for genotype, treatment and genotype-by-treatment interaction (Table S3). The effects of treatment and genotypes were highly significant at all time points. Genotype-by-treatment interactions were more pronounced at early time points, and no significant interaction was observed at the last time point.

**Fig. 4:**
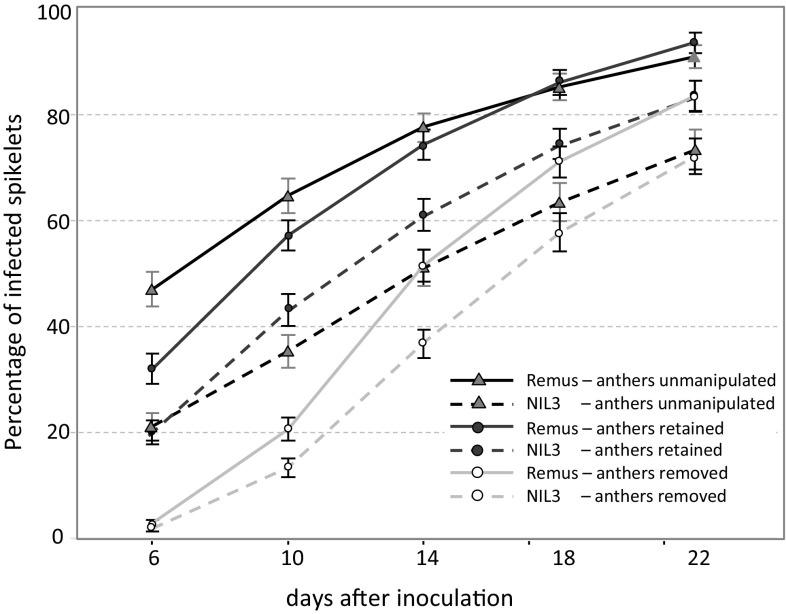
Line graphs of disease progress on inoculated heads grouped by genotype-by-treatment combination from dai(6) to dai(22). Error bars depict standard errors

**Fig. 5:**
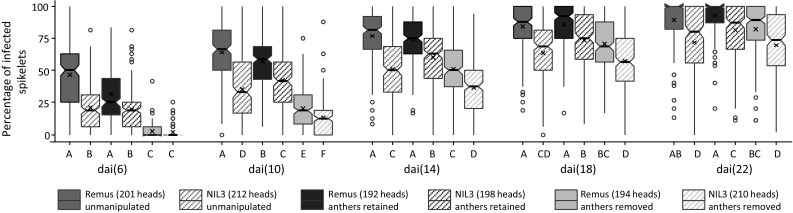
Boxplot distributions for overall FHB severity of heads grouped by genotype-by-treatment combination at 6, 10 14, 18, and 22 days after inoculation (dai). At early time points (dai(6) to dai(14)), FHB severity primarily reflects type 1 resistance; at later time points, type 1 resistance was confounded by fungal spreading (type 2 resistance). Numbers of analyzed heads are given in the figure legend. Solid bold lines and crosses indicate the medians and the means, respectively. Open circles represent outliers. Means of groups with different letters are significantly different at *p* < 0.01 using Tukey’s multiple range test

Disease progress curves of FHB severity for all analyzed genotype-by-treatment combinations are depicted in Fig. 4. Unmanipulated variants of Remus and NIL3 differed significantly at all time points, and these differences were constant in the range of 20 to 30% less symptomatic spikelets for NIL3. Remus and NIL3 were at all time points clearly more resistant when simulating complete anther extrusion compared to their respective variants of retained anthers, and similarly, removing anthers improved resistance in comparison with unmanipulated variants with greater differences at earlier time points and considerably higher resistance improving effects for the susceptible Remus (Table S2, Figs. 4, 5).

Simulating complete anther retention in the resistant NIL3 clearly increased FHB severity, while it had no effect on the resistance level or even increased resistance (dai(6), dai(10)) in Remus compared to unmanipulated heads. Notably, NIL3 retained was still less diseased than Remus retained, and these differences were stable and ranged from 10 to 14% between time points. At early time points, complete anther extrusion established high resistance for both genotypes, with NIL3 being only slightly more resistant than Remus, followed by NIL3 unmanipulated and NIL3 retained. At the late time points, the ranking changed slightly and NIL3 removed and NIL3 unmanipulated were the most resistant variants, followed by Remus removed and NIL3 retained. Remus retained and Remus unmanipulated were most susceptible at all time points. The rank order was very stable among experiments (Fig. 4, Table S2).

## Discussion

Buerstmayr et al. (2002, 2003) reported the major type 1 resistance QTL *Qfhs.ifa*-*5A* that mapped across the centromere of chromosome 5A. Type 1 resistance is difficult to assess as it is confounded by type 2 resistance and the environment, especially by weather condition at anthesis (Rudd et al. 2001). In the current study, the NI-RIL population was fixed for *Fhb1* in order to reduce spreading of the disease and confounding with type 2 resistance. Flowering time was very homogenous and took only a few days so that experiments were not affected by changing weather conditions during flowering period (data not shown). High heritability and high correlation coefficients between experiments affirmed reliable and reproducible phenotypic data.

### Fine-mapping of *Qfhs.ifa*-*5A*

Numerous QTL mapping studies detected resistance QTL derived from several other germplasms that overlapped with *Qfhs.ifa*-*5A* highlighting the pericentromeric region of chromosome 5A as a hot spot for Fusarium resistance genes. Unfortunately, recombination around centromeres is highly suppressed and this effect increases toward the centromere, where recombination is almost absent (Akhunov et al. 2003; Lukaszewski and Curtis 1993). This makes fine-mapping of *Qfhs.ifa*-*5A* extremely difficult. However, out of 3650 F_2_ NILs, seventy were found to recombine in the target interval, and genotyping these NI-RILs partitions the initial QTL interval into 12 bins (Buerstmayr et al. 2018). All NILs have more than 98% of their genetic background fixed for the recurrent parent; hence, no interference of other QTL is expected and a clear discrimination between resistant and susceptible NILs is assumed. Frequency distributions of FHB severity, FHB incidence and AR were bimodal (Fig. 1) although a few NILs constantly showed intermediate phenotypes in all experiments and could thus neither be classified as clearly resistant nor as clearly susceptible genotypes. QTL analysis separated the initially reported single QTL into two linked QTL: a major effect QTL that mapped across the centromere, and a minor effect QTL positioned at the distal half of the short arm of chromosome 5A (Table 2, Fig. 2). Although both *Qfhs.ifa*-*5Ac* and *Qfhs.ifa*-*5AS* were delimited to genetic intervals as small as 0.1 and 0.2 cM, respectively, the corresponding physical distances were large and comprised approximately 44.1 and 49.2 Mbp. In 2011, Xue and associates fine-mapped *Fhb5* to the C-5AS3-0.75 bin on the 5A short arm between *gwm415* and *gwm304* (Xue et al. 2011). Due to similarities between *Fhb5* and *Qfhs.ifa*-*5A*—both QTL confer type 1 resistance, both have their resistance derived from the Asian gene pool and both mapped to the pericentromeric region of chromosome 5A—we initially assumed that *Fhb5* and *Qfhs.ifa*-*5A* possibly share the same resistance gene. However, comparing QTL positions revealed that *Fhb5* was located in between *Qfhs.ifa*-*5AS* and *Qfhs.ifa*-*5Ac* pointing toward independent genetic controls for *Fhb5* and *Qfhs.ifa*-*5A*. More recently, Jia et al. (2018) summarized current knowledge of FHB resistance in the Chinese wheat landrace Wangshuibai and an updated refined map placed *Fhb5* to a 0.09 cM interval very close to the centromere and therefore partially overlapped with *Qfhs.ifa*-*5Ac* proposing a common resistance gene for the two QTL. According to our mapping results, the arm location of the *Qfhs.ifa*-*5Ac* underlying resistance gene is still undetermined.

### The associations of *Qfhs.ifa*-*5Ac* and *Qfhs.ifa*-*5AS* with anther retention and plant height

The parental lines NIL1 and NIL2 not only had clearly distinct resistance levels; they also differed widely in retaining anthers (Table 1) suggesting that *Qfhs.ifa*-*5A* might also affect AR. And indeed a high phenotypic correlation of AR to FHB severity and incidence was observed; moreover, two QTL for AR were identified that coincided with *Qfhs.ifa*-*5AS* and *Qfhs.ifa*-*5Ac* (Figs. 2, 3, Tables 2, 3). The results indicate that anther extrusion indirectly increases type 1 resistance and supports the assumption made by Lu et al. (2013) that several of the many published FHB resistance QTL could actually be caused by increased anther extrusion. Among the few mapping studies that included anthers as a possible resistance component, most QTL for anther extrusion were found to coincide with QTL for FHB severity traits (Buerstmayr and Buerstmayr 2015, 2016; Lu et al. 2013; Skinnes et al. 2010). This strongly suggests a pleiotropic effect of anther extrusion on FHB resistance. Factors that may influence AR are manifold: Size and architecture of spikes and spikelets, tenacity and form of glumes, tissue structure, lodicule swelling, size of anthers and length of filaments, flower opening width and flower opening duration all potentially affect AR and its complement anther extrusion. Unsurprisingly, the genetic control of AR was found to be highly complex and under polygenic control. Despite high heritability coefficients, primarily small to medium effect QTL have been identified (Boeven et al. 2016; Buerstmayr and Buerstmayr 2015; Lu et al. 2013; Muqaddasi et al. 2017a, b; Skinnes et al. 2010) and solely the *Rht1* genes have been repeatedly associated with AR (Boeven et al. 2016; Buerstmayr and Buerstmayr 2016; Lu et al. 2013; Wurschum et al. 2018). *Qfhs.ifa*-*5A* strongly influences the proportion of extruded anthers. As anther extrusion is an important pollinator trait, introgression *Qfhs.ifa*-*5A* can simultaneously improve FHB resistance and pollinator traits that are essential for hybrid breeding.

Although parental NILs did not differ in PH, variation was present in the NI-RIL population and a moderate negative correlation of PH to FHB severity and incidence was found. Furthermore, two QTL linked in repulsion were identified and coincided with *Qfhs.ifa*-*5AS* and *Qfhs.ifa*-*5Ac.* The resistance allele at *Qfhs.ifa*-*5Ac* increased PH, while it decreased PH at *Qfhs.ifa*-*5AS*. As QTL acted additively, only small differences in PH were observed for genotypes having either both or neither of the two resistance QTL (Figs. 2, 3, Tables 2, 3). The effects on PH were only minor, and NILs contrasting at both QTL differed on average by ~ 5 cm only. It is well recognized that taller plants tend to be more resistant. PH potentially influences microclimate such as relative humidity, leaf wetness duration, and temperature at spike height as shorter plants are more affected by soil humidity and dew and a denser canopy structure may lead to reduced air circulation (Buerstmayr and Buerstmayr 2016; Hilton et al. 1999; Jones 1983). High humidity and temperature promote infection and disease development. As such, PH contributes as a passive resistance factor for FHB. However, whether and to what extent a height difference of 5 cm influenced FHB resistance is unclear. PH is a quantitative trait; modulated by a few large and many small effect QTL (Mao et al. 2010; Snape et al. 1977). From the current study, it remains unclear whether PH and AR are under a common genetic control or tightly linked genes are the reason of the simultaneous effect on both traits at *Qfhs.ifa*-*5Ac* and *Qfhs.ifa*-*5AS*. Considering the gene content in the Chinese Spring reference sequence assembly of the corresponding QTL intervals at *Qfhs.ifa*-*5Ac* and *Qfhs.ifa*-*5AS* (101 HC plus 248 LC genes, 240 HC plus 351 LC), it appears likely that genes effecting PH as well as genes effecting AR are present, but due to repressed recombination, it was probably impossible to isolate PH QTL from AR QTL.

### Anthers and their association with FHB resistance

The results of fine-mapping *Qfhs.ifa*-*5A* as well as the greenhouse experiments emphasize the relevance of anthers for FHB resistance. Through simulating complete anther extrusion, both, resistant NIL3 (carrier of *Qfhs.ifa*-*5A*) and susceptible Remus became almost equally resistant at early time points after inoculation. Both lines were significantly less diseased than their unmanipulated control heads, whereas the increase in resistance was much smaller for NIL3 with inherent higher anther extrusion than Remus. At the later time points, the positive effect of anther removal on FHB resistance became smaller for Remus and disappeared completely for NIL3 (Fig. 4). Results of our experiments together with earlier studies clearly show that anthers are not necessarily required for a successful infection (Schroeder 1955; Tu 1930). In accordance with our results, pollen and anthers were found to be the preferred targets in the initial stages of infection for both resistant and susceptible cultivars (Miller et al. 2004). Although the absences of anthers effectively reduced initial infection and early disease development, it did not protect plants from fungal spreading within the spikes.

Manually retaining anthers in NIL3 significantly increased susceptibility, while it did not increase susceptibility in the cultivar Remus; moreover, mimicking complete anther retention in Remus even improved resistance compared to unmanipulated heads. Remus control heads had inherently many anthers retained, with most of these anthers trapped and partially exposed between palea and lemma, so that the florets could not properly close after pollination, thereby providing an easy entry for the fungus. Our manual procedure to pretend anther retention separated anthers from filaments and placed them back into the florets; as a result, anthers were mostly caught inside the floral cavity, fewer were trapped between palea and lemma and most florets could properly close after pollination and thereby hamper fungal colonization. These facts may potentially explain the higher susceptibility of the unmanipulated Remus compared to the manually retained variants. This is in line with the observations made by Kubo et al. (2010, 2013a) that genotypes with partially exposed anthers were more diseased than cleistogamous, closed flowering genotypes and genotypes with high levels of anther extrusion. Long flowering duration and wide flower opening also increased the risk of FHB infection (Gilsinger et al. 2005). Once the spores have successfully entered the florets, the floral cavities provide optimal conditions for the pathogen development. Microscopic observations revealed that spore germination and growth of the hyphal network was much more evident in the presence of anthers, pollen and stigma while colonization progressed more slowly on the harder tissues of the lemma and palea (Kang and Buchenauer 2000; Miller et al. 2004; Pugh et al. 1933). As no evidence has been found so far that endogenous compounds in floral parts, including anthers and pollen, may affect infection and tissue colonization (Engle et al. 2004), we hypothesize that the fragile and easily decaying tissue structure of anthers potentially offers little resistance to the fungus and thus may be a preferred target for initial infection promoting the establishment of the fungus.

Notably, with complete anther retention, NIL3 was never as susceptible as Remus and pretending complete anther extrusion NIL3 was—except for the earliest time point—always more resistant then the respective Remus variants. As NIL3 and Remus differ only for the *Qfhs.ifa*-*5A* interval it is likely that—in addition to the passive resistance provided by increased anther extrusion—other resistance components are present at *Qfhs.ifa*-*5A*.

## Conclusion and future perspectives

*Qfhs.ifa*-*5A* is one of the best validated FHB resistance QTL, it has been introgressed in adapted breeding material showing consistent, strong reduction of disease severity—the responsible resistance mechanisms/genes behind the QTL have not yet been characterized. Fine-mapping *Qfhs.ifa*-*5A* revealed the underlying genetic control of the FHB resistance as highly complex. The strong resistance improving effect of *Qfhs.ifa*-*5A* is based on two tightly linked QTL both affecting resistance against initial infection, anther extrusion and to a lesser extent PH. Simulating complete anther extrusion in two genotypes differing only in *Qfhs.ifa*-*5A* suggests that resistance conferred by *Qfhs.ifa*-*5A* can be explained by a higher proportion of extruded anthers and proposes a pleiotropic dependency of the two traits. Furthermore, results indicate that additional resistance factor(s) at the QTL may be responsible for the consistently lower disease severities of the *Qfhs.ifa*-*5A* carrier compared to the recurrent parent when establishing same extents of anther extrusion/retention for both genotypes.

The knowledge of anther extrusion as a major resistance component of *Qfhs.ifa*-*5A* will assist in gene identification with still numerous genes present in the fine-mapped QTL intervals by suggesting candidates for further analysis/reverse genetics. A detailed investigation of individual factors that influence the extent of anther extrusion, such as anther size and filament length, swelling of lodicules, flower opening width and duration, glume stiffness can help elucidating the very complex resistance of *Qfhs.ifa*-*5A* and support gene cloning.

For practical breeding application—at least two genes in coupling phase responsible for resistance and their positions in the low-recombinogenic centromeric region support introgression of the complete resistance locus. A higher proportion of extruded anthers as the main mechanism behind the QTL enables fast and reliable phenotypic selection even without disease pressure, making *Qfhs.ifa*-*5A* very attractive for deployment in practical resistance breeding programs.

## Electronic supplementary material

Below is the link to the electronic supplementary material.

Supplementary material 1 (PDF 302 kb)

Supplementary material 2 (PDF 519 kb)

Supplementary material 3 (XLSX 21 kb)

Supplementary material 4 (PDF 282 kb)
