# Supplementary material for: Fine-mapping of the Fusarium head blight resistance QTL Qfhs.ifa-5A identifies two resistance QTL associated with anther extrusion
Source: Theor Appl Genet. 2019 Apr 4;132(7):2039–53. doi: 10.1007/s00122-019-03336-x (PMC6588648; doi:10.1007/s00122-019-03336-x)
Supplement: Supplementary file 1 — Supplementary material 1 (PDF 302 kb) [file 122_2019_3336_MOESM1_ESM.pdf]

Online Resource 1

Article title: Fine-mapping of the Fusarium head blight resistance QTL *Qfhs.ifa-5A* identifies two resistance QTL associated with anther extrusion

Journal: Theoretical and Applied Genetics

Authors: Barbara Steiner, Maria Buerstmayr, Christian Wagner, Andrea Danler, Babur Eshonkulov, Magdalena Ehn, Hermann Buerstmayr

Name, affiliation, and email of corresponding author:

Maria Buerstmayr, Department for Agrobiotechnology Tulln, BOKU-University of Natural Resources and Life Sciences-Vienna, Konrad Lorenz Str. 20, 3430 Tulln, Austria

e-mail: maria.buerstmayr@boku.ac.at

TABLE S1.1 Pearson correlation coefficients among traits for individual experiments and across all experiments for FHB severity (AUDPC), FHB incidence (Inc%), anther retention (AR%) and plant height (PH)

Experiment	AUDPC:Inc%		AUDPC:AR%		Inc%:AR%		AUDPC:PH		Inc%:PH		AR%:PH	
	r	*p*	r	*p*	r	*p*	r	*p*	r	*p*	r	*p*
2014	0.91	<.0001	0.75	<.0001	0.79	<.0001	-0.32	0.0086	-0.35	0.0032	-0.33	0.0065
2015	0.89	<.0001	0.83	<.0001	0.81	<.0001	--	--	--	--	--	--
2016	0.87	<.0001	0.76	<.0001	0.74	<.0001	-0.39	0.0006	-0.38	0.0008	-0.35	0.0021
2017	--	--	0.78	<.0001	--	--	-0.21	0.07	--	--	-0.13	0.2488
overall mean	0.90	<.0001	0.88	<.0001	0.87	<.0001	-0.43	<.0001	-0.44	<.0001	-0.42	0.0002

TABLE S1.2 Pearson correlation coefficients among experiments for FHB severity (AUDPC), FHB incidence (%), anther retention (%) and plant height

Experiment	2014:2015		2014:2016		2014:2017		2015:2016		2015:2017		2016:2017	
Trait	r	*p*	r	*p*	r	*p*	r	*p*	r	*p*	r	*p*
FHB severity (AUDPC)	0.86	<.0001	0.88	<.0001	0.81	<.0001	0.89	<.0001	0.87	<.0001	0.87	<.0001
FHB incidence (%)	0.76	<.0001	0.75	<.0001	--	--	0.85	<.0001	--	--	--	--
Anther retention (%)	0.73	<.0001	0.78	<.0001	0.75	<.0001	0.69	<.0001	0.77	<.0001	0.71	0.0007
Plant height	--	--	0.46	<.0001	0.40	<.0001	--	--	--	--	0.40	0.0003
