# Supplementary material for: Fine-mapping of the Fusarium head blight resistance QTL Qfhs.ifa-5A identifies two resistance QTL associated with anther extrusion
Source: Theor Appl Genet. 2019 Apr 4;132(7):2039–53. doi: 10.1007/s00122-019-03336-x (PMC6588648; doi:10.1007/s00122-019-03336-x)
Supplement: Supplementary file 2 — Supplementary material 2 (PDF 519 kb) [file 122_2019_3336_MOESM2_ESM.pdf]

Online Resource 2

Article title: Fine-mapping of the Fusarium head blight resistance QTL *Qfhs.ifa-5A* identifies two resistance QTL associated with anther extrusion

Journal: Theoretical and Applied Genetics

Authors: Barbara Steiner, Maria Buerstmayr, Christian Wagner, Andrea Danler, Babur Eshonkulov, Magdalena Ehn, Hermann Buerstmayr

Name, affiliation, and email of corresponding author:

Maria Buerstmayr, Department for Agrobiotechnology Tulln, BOKU-University of Natural Resources and Life Sciences-Vienna, Konrad Lorenz Str. 20, 3430 Tulln, Austria

e-mail: maria.buerstmayr@boku.ac.at

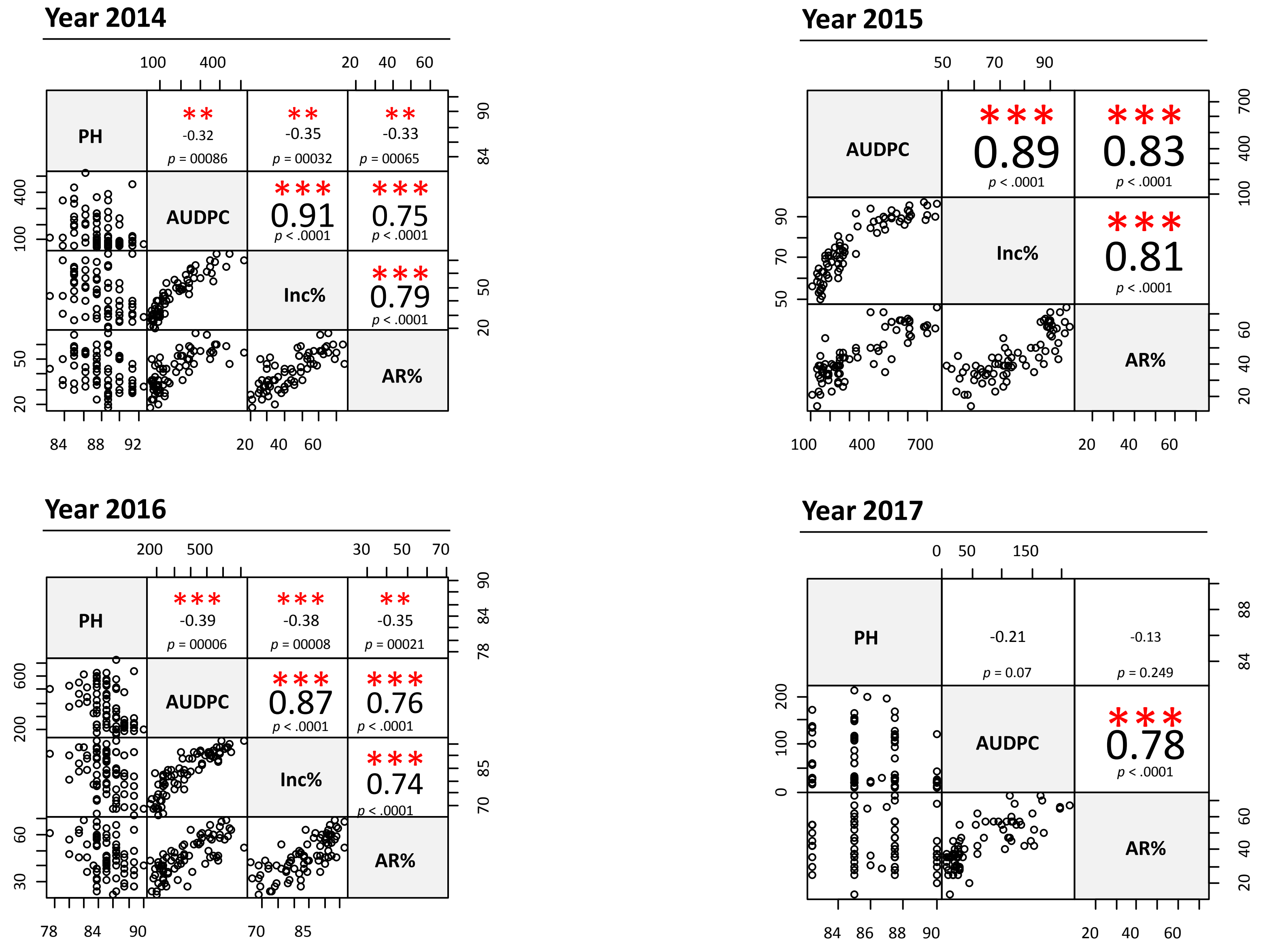

Figure S1.1 Pairwise scatter plot matrix and correlation coefficients between traits in individual years for plant height (PH), FHB severity (AUDPC), FHB incidence (Inc%) and anther retention (AR%). Pairwise scatter plots are in lower triangle boxes, and upper triangle boxes give the corresponding Pearson correlation coefficients and *p* values between variables. Diagonal boxes denote analysed traits.

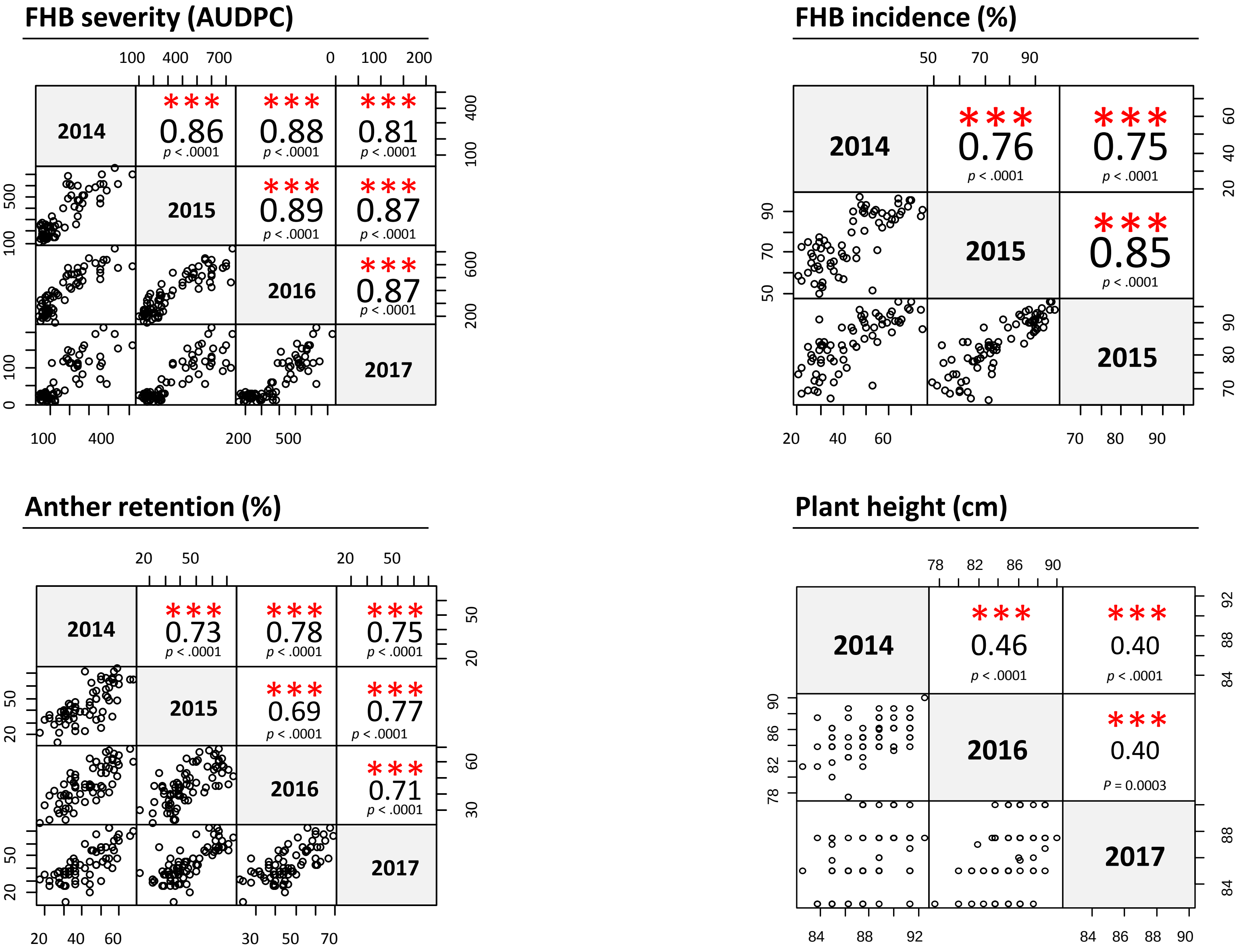

Figure S1.2 Pairwise scatter plot matrix and correlation coefficients between experiments for FHB severity, FHB incidence, anther retention and plant height. Pairwise scatter plots are in lower triangle boxes, and upper triangle boxes give the corresponding Pearson correlation coefficients and *p* values between variables. Diagonal boxes denote years.
