# Supplementary material for: Fine-mapping of the Fusarium head blight resistance QTL Qfhs.ifa-5A identifies two resistance QTL associated with anther extrusion
Source: Theor Appl Genet. 2019 Apr 4;132(7):2039–53. doi: 10.1007/s00122-019-03336-x (PMC6588648; doi:10.1007/s00122-019-03336-x)
Supplement: Supplementary file 4 — Supplementary material 4 (PDF 282 kb) [file 122_2019_3336_MOESM4_ESM.pdf]

## Online Resource 4

**Article title:** Fine-mapping of the Fusarium head blight resistance QTL *Qfhs.ifa-5A* identifies two resistance QTL associated with anther extrusion

**Journal:** Theoretical and Applied Genetics

**Authors:** Barbara Steiner, Maria Buerstmayr, Christian Wagner, Andrea Danler, Babur Eshonkulov, Magdalena Ehn, Hermann Buerstmayr

**Name, affiliation, and email of corresponding author:**

Maria Buerstmayr, Department for Agrobiotechnology Tulln, BOKU-University of Natural Resources and Life Sciences-Vienna, Konrad Lorenz Str. 20, 3430 Tulln, Austria e-mail: maria.buerstmayr@boku.ac.at

**TABLE S3** Analysis of variance of anther experiments for FHB severity measured 6, 10, 14, 18, and 22 days after inoculation (dai) for the fixed effects genotype, treatment and genotype-by-treatment interaction

| | Mean square | *F* value | *p*-value |
|---|---|---|---|
| FHB severity dai(6) | | | |
| genotype | 25781.0 | 111.3 | 0.002 |
| treatment | 11636.0 | 50.3 | <0.001 |
| genotype-by-treatment | 14362.0 | 62.0 | <0.001 |
| error | 231.6 | | |
| FHB severity dai(10) | | | |
| genotype | 42526.0 | 135.1 | <0.001 |
| treatment | 116577.0 | 370.35 | <0.001 |
| genotype-by-treatment | 11545.0 | 36.68 | <0.001 |
| error | 314.8 | | |
| FHB severity dai(14) | | | |
| genotype | 60303.0 | 160.5 | <0.001 |
| treatment | 58821.0 | 156.6 | <0.001 |
| genotype-by-treatment | 4714.0 | 12.5 | <0.001 |
| error | 375.7 | | |
| FHB severity dai(18) | | | |
| genotype | 41012.0 | 110.4 | <0.001 |
| treatment | 19332.0 | 52.0 | <0.001 |
| genotype-by-treatment | 2285.0 | 6.2 | 0.023 |
| error | 371.6 | | |
| FHB severity dai(22) | | | |
| genotype | 26028.3 | 75.7 | <0.001 |
| treatment | 7880.0 | 22.9 | 0.001 |
| genotype-by-treatment | 900.3 | 2.6 | 0.128 |
| error | 343.8 | | |
